# eDNA metabarcoding reveals biodiversity and depth stratification patterns of dinoflagellate assemblages within the epipelagic zone of the western Coral Sea

**DOI:** 10.1186/s12862-024-02220-7

**Published:** 2024-03-26

**Authors:** Megan Carve, Tahnee Manning, Aidyn Mouradov, Jeff Shimeta

**Affiliations:** School of Science, RMIT University, Melbourne, VIC Australia

**Keywords:** Amplicon sequence variants, High throughput sequenci, Great Barrier Reef, Nutritional strategies, Metabolism strategies, Protistan diversity

## Abstract

**Background:**

Dinoflagellates play critical roles in the functioning of marine ecosystems but also may pose a hazard to human and ecosystem health by causing harmful algal blooms (HABs). The Coral Sea is a biodiversity hotspot, but its dinoflagellate assemblages in pelagic waters have not been studied by modern sequencing methods. We used metabarcoding of the 18 S rRNA V4 amplicon to assess the diversity and structure of dinoflagellate assemblages throughout the water column to a depth of 150 m at three stations in the Western Coral Sea. Additionally, at one station we compared metabarcoding with morphological methods to optimise identification and detection of dinoflagellates.

**Results:**

Stratification of dinoflagellate assemblages was evident in depth-specific relative abundances of taxonomic groups; the greatest difference was between the 5–30 m assemblages and the 130–150 m assemblages. The relative abundance of Dinophyceae (photosynthetic and heterotrophic) decreased with increasing depth, whereas that of Syndiniales (parasitic) increased with increasing depth. The composition of major taxonomic groups was similar among stations. Taxonomic richness and diversity of amplicon sequence variants (ASVs) were similar among depths and stations; however, the abundance of dominant taxa was highest within 0–30 m, and the abundance of rare taxa was highest within 130–150 m, indicating adaptations to specific depth strata. The number of unclassified ASVs at the family and species levels was very high, particularly for Syndinian representatives.

**Conclusions:**

Dinoflagellate assemblages in open water of the Coral Sea are highly diverse and taxonomically stratified by depth; patterns of relative abundance along the depth gradient reflect environmental factors and ecological processes. Metabarcoding detects more species richness than does traditional microscopical methods of sample analysis, yet the methods are complementary, with morphological analysis revealing additional richness. The large number of unclassified dinoflagellate-ASVs indicates a need for improved taxonomic reference databases and suggests presence of dinoflagellate-crypto and–morphospecies.

**Supplementary Information:**

The online version contains supplementary material available at 10.1186/s12862-024-02220-7.

## Background

Dinoflagellates are ubiquitous in marine environments and have essential roles in ecological interactions that shape ecosystems and affect human societies [1–3]. They are most known for the role of specific dinoflagellate taxa in causing harmful algal blooms (HABs) [4–6]. Dinoflagellates utilize numerous nutritional strategies, including phototrophy, heterotrophy, and mixotrophy [1, 2], and have a fundamental role in marine food webs and biogeochemical cycling. Along with other phytoplankton, photosynthetic dinoflagellates contribute up to 70% of oxygen in the atmosphere [7]. Describing the taxonomic and functional diversity of dinoflagellates is essential to understanding their ecological roles and interactions in marine ecosystems. To this end, DNA metabarcoding, which facilitates the identification of multitudes of species simultaneously by sequencing the DNA isolated from the environment (eDNA), offers advantages over classical identification methods based on morphological analysis in large-scale diversity assessments [8].

Metabarcoding is increasingly being used to further our understanding of species assemblages and spatial distribution patterns of marine protists [4, 9–11]. It is especially useful for discerning rare species with low abundances, and taxa that are difficult to identify morphologically using classical methods [3, 12–14]. Metabarcoding is proving particularly useful for detecting and monitoring HAB taxa and improving models of HAB prediction [13, 15–19]. Dinoflagellate studies typically target the variable regions (V1-V9, V4) or internal transcribed spacer (ITS) region of 18 S rRNA [20, 21]. Due to its popularity, a substantial number of V4 sequences have been deposited in public repositories and incorporated in taxonomic reference databases [22]. A recent advance in the application of metabarcoding in assessing dinoflagellate diversity is the development of DINOREF, a curated 18 S rRNA reference database of dinoflagellates, representing 149 genera and 422 species [23].

The Coral Sea in the southwestern Pacific Ocean harbors a diverse array of marine habitats and contains the world’s largest reef system, the Great Barrier Reef (GBR) [24]. Bordered in the west by Australia and New Guinea, on the east by New Caledonia and the New Hebrides, and on the north by the Solomon Islands, the Coral Sea covers an area of ≈ 4,700,000 km^2^ with a maximum depth of 9,140 m (average depth ≈ 2,400 m) [24, 25]. The geomorphic features of the Coral Sea include abyssal plains, plateaus, slopes, undersea canyons, volcanic seamounts, and deep ocean trenches. Interactions between these geomorphic features and ocean currents cause upwelling of nutrient-rich water that drives regional productivity and contributes to the formation of distinct ecological communities [25, 26]. The Coral Sea is a recognized biodiversity hotspot, supporting a high biodiversity of cetaceans [27], sharks [28], fish [26, 29], and micronektonic species [30, 31].

Studies on dinoflagellate diversity and distribution in the Coral Sea are limited to non-metabarcoding approaches (e.g. morphological identification, cell size, chlorophyll content) that have examined the wider phytoplankton and marine protist assemblages in near-surface waters in coastal regions of the GBR [32–35]. Dinoflagellate induced HABs are an important indicator of ecosystem health and have potential to contaminate fisheries operating in the Coral Sea [36–39]. Enhancing our understanding of the taxonomic and functional diversity of dinoflagellates in the Coral Sea can have important ecological, social, and economic outcomes. To date, the diversity and structure of dinoflagellate assemblages and their latitudinal and vertical distribution patterns in the open-ocean water masses in the Coral Sea remain largely unexplored. To address this knowledge gap, we employed eDNA metabarcoding of the V4 region of the 18 S ribosomal RNA gene and morphology-based identification methods to characterize the dinoflagellate assemblage and its vertical distribution from depths spanning 0–150 m at three stations in the Western Coral Sea, yielding novel insights into depth-dependence of assemblage structure.

## Results

General summary of amplicons produced by the DADA2 pipeline.

After the filtering, denoising, merging, and chimera removal steps undertaken with the DADA2 pipeline, 3,436,037 reads remained which were classified as 9,560 unique amplicon sequence variants (ASVs). After removing ASVs not assigned to a division and ASVs that contained one sequence within the entire dataset, 7,871 ASVs (3,369,477 reads) remained. Rarefaction curves showed that sampling was sufficient to capture diversity in each sample (Supplementary File 1, Figure S1).

**Fig. 1:**
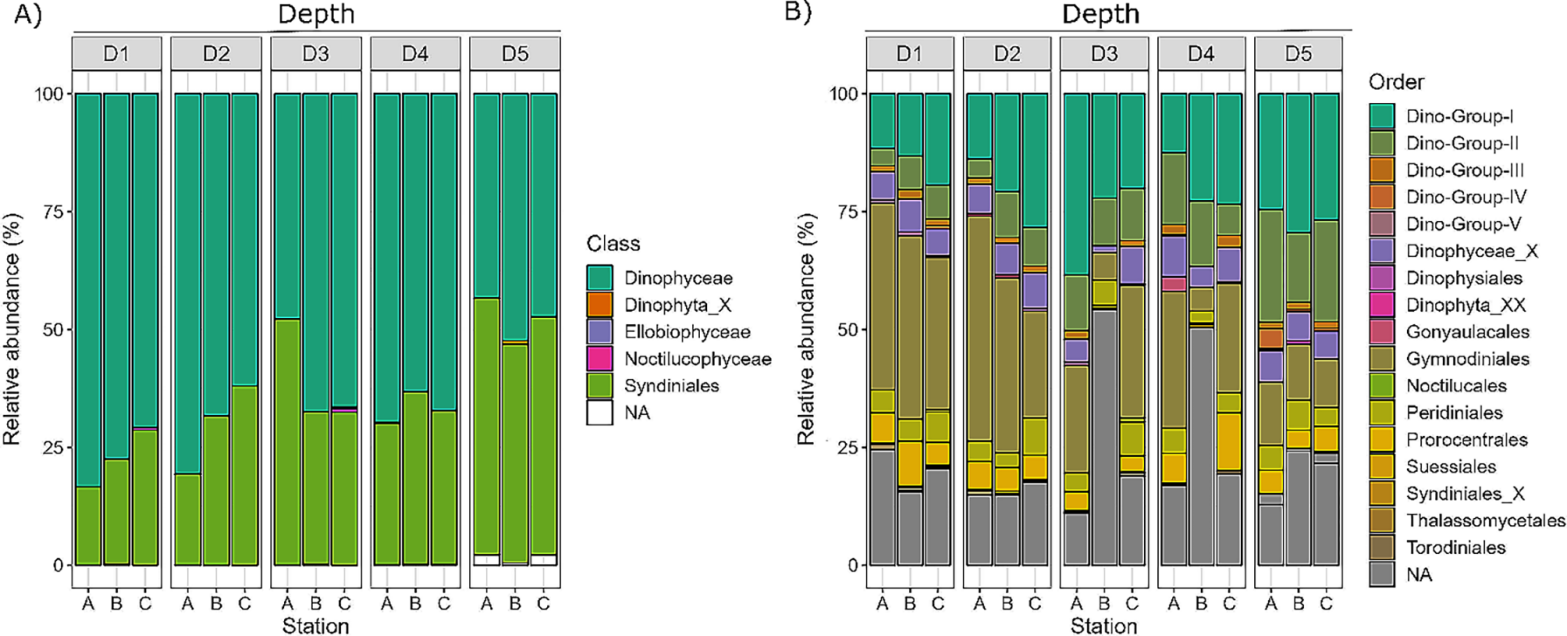
Relative abundances of dinoflagellate ASVs throughout the water column to a depth of 150 m beneath the sea surface at depth zones (D1: 5–10 m, D2: 20–30 m, D3: 45–60 m, D4: 95–120 m, D5: 130–150 m) at Station A, B, and C. Colours represent major dinoflagellate taxonomic groups at the level of: (**A**) class, and (**B**) order

**Fig. 2:**
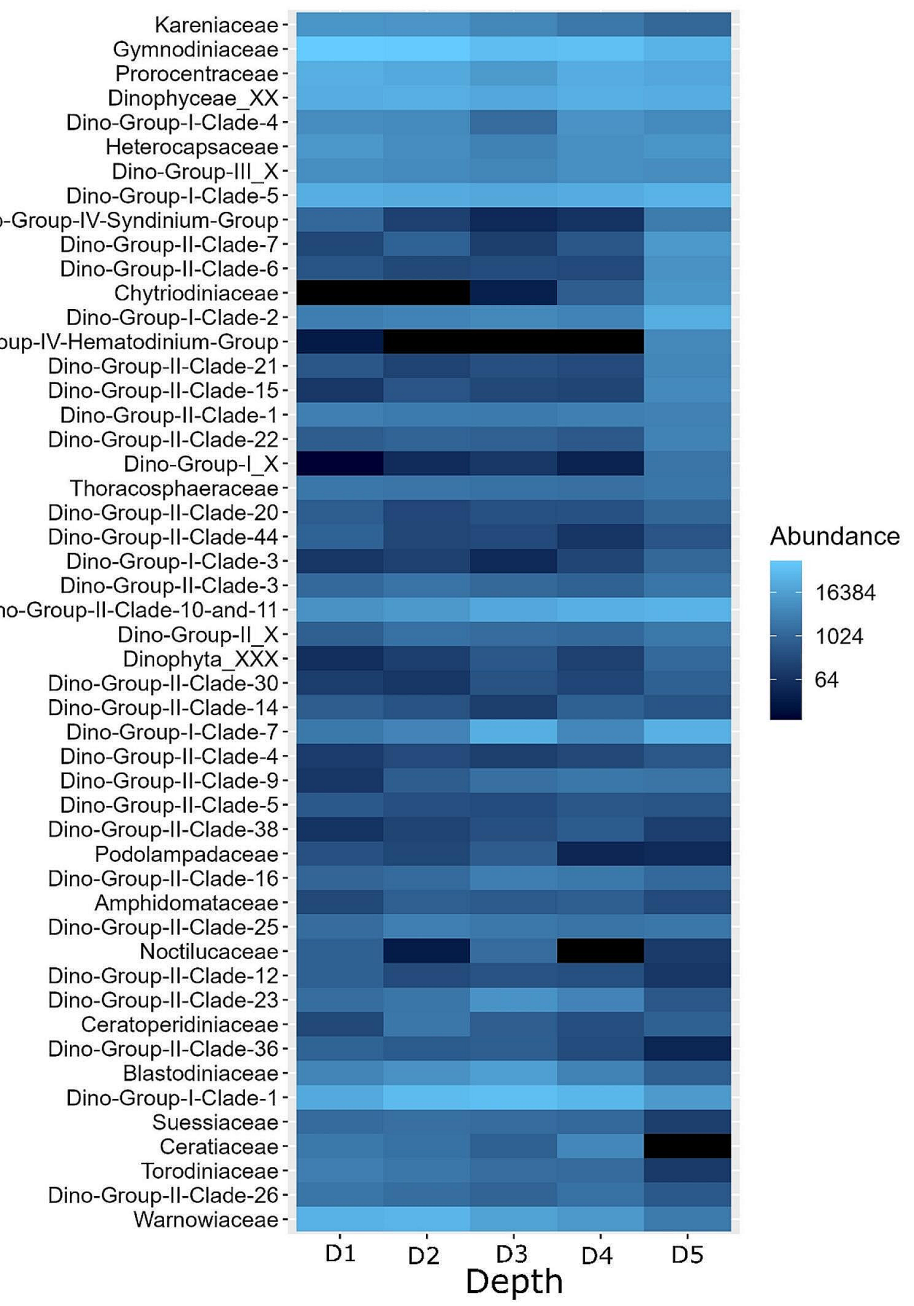
Heatmap showing family-level abundance of dinoflagellate ASVs throughout the water column to a depth of 150 m beneath the sea surface at depth zones (D1: 5–10 m, D2: 20–30 m, D3: 45–60 m, D4: 95–120 m, D5: 130–150 m) at Station A, B and C

Across all stations, Dinoflagellata ASVs were affiliated with 4 classes, 14 orders, 76 families, and 51 genera, and 63 species (Fig. 1A&B, Supplementary File 2). Supplementary File 1, Table S1, provides a list of identified species and their functional classification. Unclassified ASVs were present at each taxonomic rank. Dinoflagellata was mainly represented by ASVs affiliated with class Dinophyceae (Figs. 1A and 64.5%, 2,788 ASVs). Dinophyceae was about twice as abundant as Syndiniales (34.9%, 2,235 ASVs). Dinophyceae was represented by seven orders: Gymnodiniales, Prorocentrales, Peridiniales, Gonyaulacales, Torodiniales, Suessiales, Dinophysiales, and ASVs of an undetermined order (Dinophyceae X, 6.2%, 343 ASVs) (Fig. 1B). The most abundant Dinophyceae order was Gymnodiniales (24.5%, 605 ASVs) (Fig. 1B, Supplementary File 3), which was mainly represented by ASVs affiliated with the family Gymnodiniaceae (17%, 334 ASVs) (Fig. 2).

The most abundant Syndiniales group was Dino-Group-I (21.8%, 732 ASVs) (Fig. 1B, Supplementary File 3), followed by Dino-Group-II (11.3%, 1,321 ASVs). DinoGroup-I were represented by eight clades and a group of ASVs of an undetermined clade (Dino-Group-I X). The most abundant Dino-Group-I clades were Clade-1 (8.8%, 133 ASVs), Clade-5 (4.4%, 361 ASVs), and Clade-7 (3.3%, 22 ASVs) (Fig. 2). Dino-Group-II was composed of ASVs affiliated with 41 clades and a group of ASVs of an undetermined clade (Dino-Group-II X) (Fig. 2). Most Dino-Group-II ASVs were affiliated with Dino-Group-II Clade-10-and-11 (4.8%, 230 ASVs) (Fig. 2), which was at least almost six-fold more abundant than other frequently observed DinoGroup-II clades including Clade-23 (0.8%, 28 ASVs), Clade-1 (0.7%, 212 ASVs), and Clade-7 (0.6%, 60 ASVs).

Classes Noctilucophyceae (23 ASVs) and Ellobiophyceae collectively accounted for less than 1% of the total normalized Dinoflagellata reads. Noctilucophyceae were represented by ASVs affiliated with the order Noctilucales, assigned to the family Noctilucaceae (0.09%, 14 ASVs) or Kofoidiniaceae (0.01%, 9 ASVs). Noctilucophyceae were mainly represented by *Noctiluca scintillans* (82.3% Noctilucophyceae reads, 12 ASVs). Ellobiophyceae was only represented by *Ellobiopsis chattonii* (107 reads, 3 ASVs).

Fewer taxonomic names were assigned to ASVs at each increasingly lower taxonomic rank. At the level of class, 64 out of the total 5,103 ASVs were unclassified (0.5%); whereas at the level of order, 1,691 ASVs, which represented over a quarter of dinoflagellate reads (629.2%), were unclassified. Almost a third of the dinoflagellate dataset lacked family-level classification (33.4%, 2,241 ASVs), and the majority of ASVs lacked genus-level taxonomic classifications (71.4%, 4,180 ASVs), and species level classification (89%, 4,709 ASVs).

### Comparative composition of dinoflagellate assemblages among depths and stations.

**Fig. 3:**
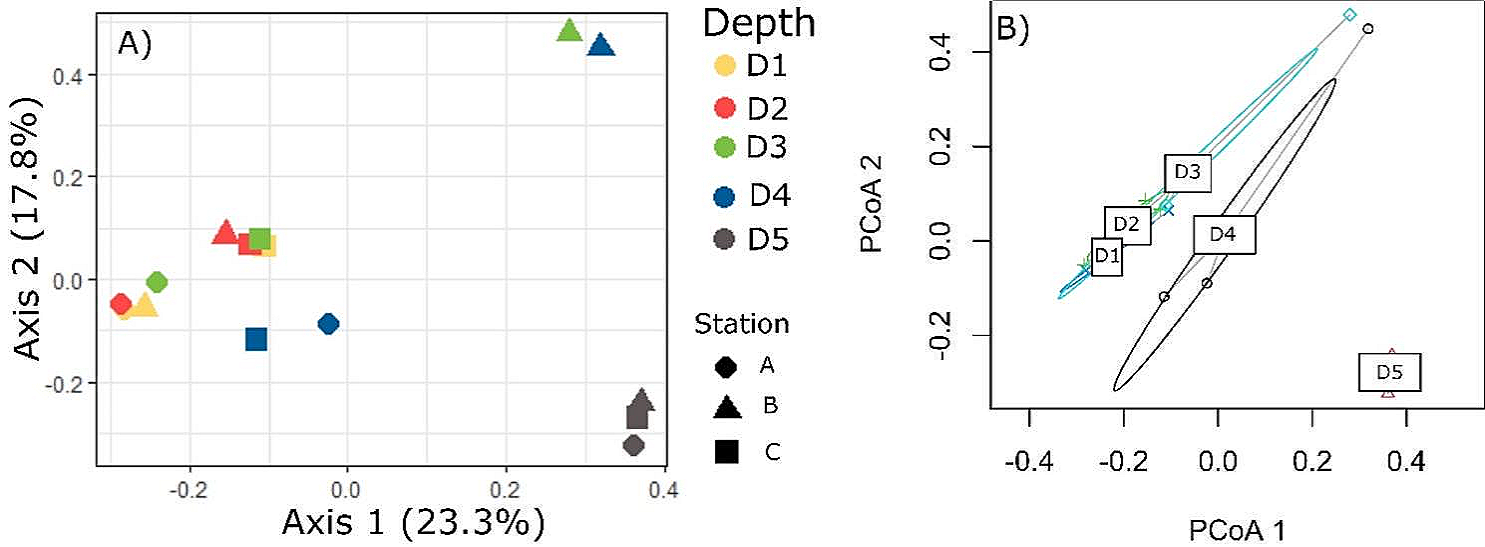
Ordination plots of Principal Coordinate Analysis (PCoA): (**A**) ASV abundance among depth zones (D1: 5–10 m, D2: 20–30 m, D3: 45–60 m, D4: 95–120 m, D5: 130–150 m) and stations (Station A, B, and C), and (**B**) multivariate homogeneity of group dispersions by depth, calculated on Bray-Curtis dissimilarity of normalized Hellinger-transformed ASV abundance data

Both PCoA and PERMANOVA analysis of Hellinger-transformed, normalized abundance data showed that the dinoflagellate assemblage differed among depths (Fig. 3; Table 1), and to a lesser extent, among stations. Two-way PERMANOVA indicated that there was a significant difference in dinoflagellate assemblage among depths (*p* = 0.003) and stations (*p* = 0.037), which explained 38.4% and 17.2% of assemblage variation, respectively. Group dispersions were homogenous for depth (Fig. 3B, *p* = 0.746) and station (*p* = 0.495). These findings show that significant variability in dinoflagellate assemblage structure occurs along depth and latitudinal gradients.

**Table 1:** PERMANOVA results for dinoflagellate assemblage alpha diversity (observed richness, Shannon Diversity, and Dominance and Rarity Indices) and assemblage structure throughout the water column to a depth of 200 m beneath the sea surface at depth zones (D1: 5–10 m, D2: 20–30 m, D3: 45–60 m, D4: 95–120 m, D5: 130–150 m) at Station A, B, and C. Alpha diversity data were raw counts. Assemblage composition data were normalized to median sequencing depth and Hellinger-transformed before analysis. Significant effects at *p* < 0.05 are highlighted in bold

Diversity metric	Factor	Df	Sum of squares	R^2^	F	p
Assemblage structure	Depth Station Residual Total	4 2 8 14	1.548 0.693 1.790 4.030	0.384 0.172 0.443 1	1.73 1.55	**0.003** **0.037**
Dominance index	Depth Station Residual Total	4 2 8 14	0.134 0.004 0.026 0.163	0.819 0.023 0.156 1	10.46 0.597	**0.004** 0.576
Rarity index	Depth Station Residual Total	4 2 8 14	0.160 0.020 0.054 0.234	0.683 0.086 0.229 1	5.96 1.51	**0.020** 0.313
Observed richness	Depth Station Residual Total	4 2 8 14	53,341 154,683 502,666 710,689	0.075 0.217 0.707 1	0.21 1.23	0.927 0.376
Shannon diversity	Depth Station Residual Total	4 2 8 14	1.069 0.701 2.058 3.829	0.279 0.183 0.537 1	1.04 1.36	0.397 0.302

Patterns of dinoflagellate distribution along depth and latitudinal gradients were evident from the PCoA based on the ASVs resemblance matrix (Fig. 3A). The dinoflagellate assemblages at D5 at stations A, B and C ordinated close to one another and were separated from the assemblages at D1– D4 in the PCoA (Fig. 3A&B). Stations A and C had relatively similar assemblages that were relatively consistent at depths D1 - D4. Station B’s assemblage was similar to the other stations at the two shallowest depth ranges (D1 - D2), but it was divergent from all other samples at intermediate depths (D3 - D4).

Indicator analysis identified 78 ASVs (of the total 5,103 ASVs) that were strongly correlated with a depth or combination of depths (Supplementary File 1, Table S3). Of these, 60 ASVs were associated with a single depth (D1 = 2 ASVs, D2 = 2 ASVs, D3 = 1 ASV, and D5 = 55 ASVs), and 18 ASVs were associated with a combination of depths (2 depths: D1 + D2 = 1 ASV, D2 + D3 = 1 ASV, D4 + D5 = 10 ASVs; 3 depths: D1 + D2 + D3 = 1 ASV, D3 + D4 + D5 = 1 ASV; 4 depths: D1 + D2 + D3 + D4 = 4 ASVs). There was a strong positive correlation for all ASVs associated with a single depth (ϕ > 0.8, Supplementary File 1, Table S3). In contrast to depth, only 10 ASVs were identified as indicators for stations (Supplementary File 1, Table S4). Of these, 9 ASVs were associated with a single station (ASVs with Station A, 1 ASV with Station B, and 2 ASVs with Station C), and there was a moderate to strong correlation for each ASV to their associated station (ϕ > 0.7–0.87, Supplementary File 1, Table S4). In addition, one ASV was associated with two Stations (Stations A + C, ϕ = 0.76). The taxonomic resolution of indicator ASVs was typically at the level of the family.

Across all stations, distribution patterns of the major taxonomic groups varied along the depth gradient (Figs. 1 and 2). The abundance of class Dinophyceae tended to decrease with increasing depth throughout the water column (Fig. 1A); but was similar at D4 and D2. This depth-associated pattern was mainly driven by the abundance pattern of order Gymnodiniales (Fig. 1B). Gymnodiniales was over three-fold as abundant at D1 and D2 (7.4% and 7.2%, respectively) than at D5 (2.4%) and was evenly distributed at D3 and D4 (3.4% and 3.8%, respectively). The abundances of Gymnodiniales families Warnowiaceae, Kareniaceae, and Gymnodiniaceae decreased with increasing depth (Fig. 2); in contrast, Ceratoperidiniaceae and Chytriodiniaceae did not follow this pattern. Chytriodiniaceae was most abundant at D5 (4.3%) and absent at D1 and D2 (Fig. 2); Ceratoperidiniaceae was more abundant at D2 (0.1%) than at other depths (0.02 ± 0.01%).

### Alpha diversity

**Fig. 4:**
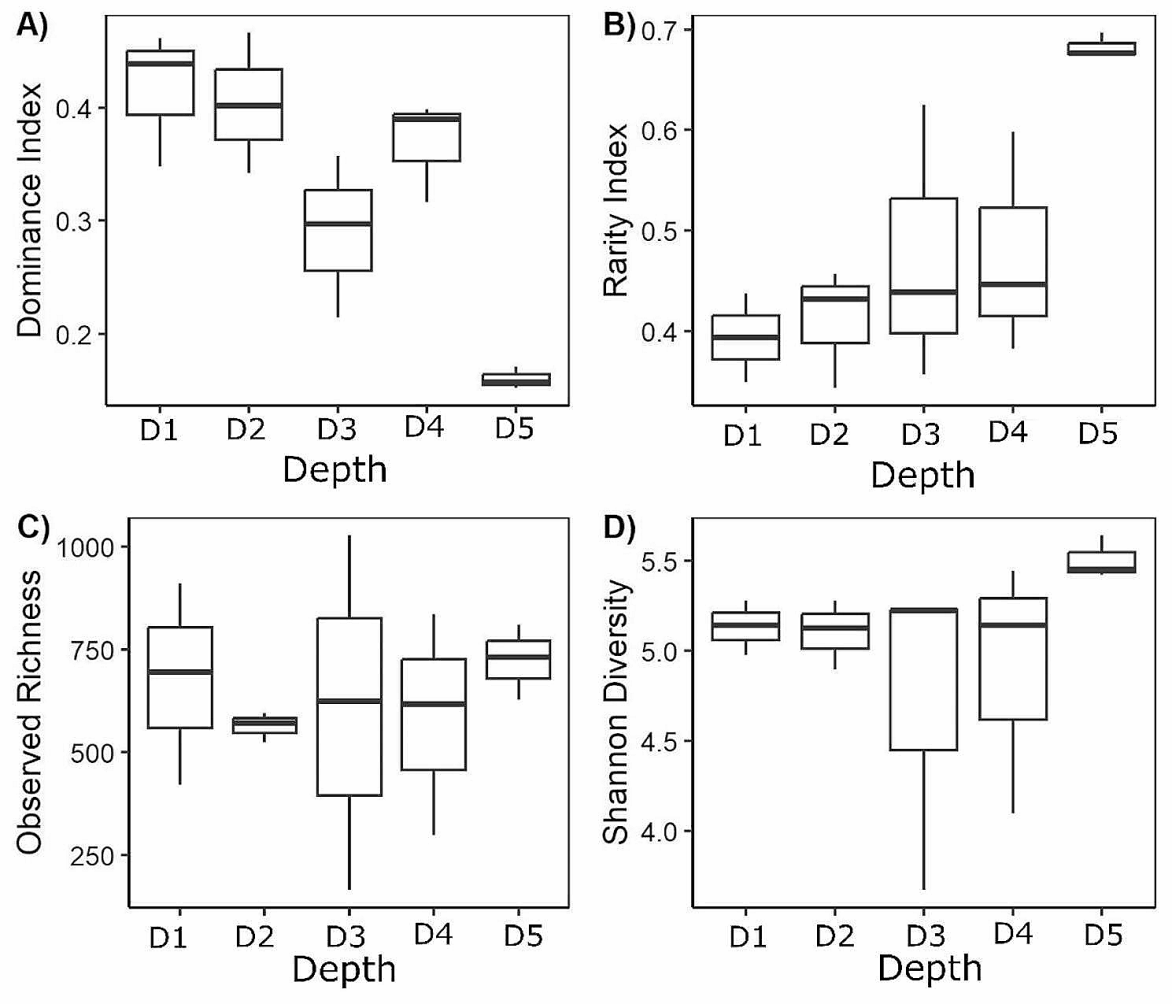
Comparison of dinoflagellate assemblage alpha diversity at depth zones (D1: 5–10 m, D2: 20–30 m, D3: 45–60 m, D4: 95–120 m, D5: 130–150 m) at Station A, B, and C. (**A**) Dominance Index, (**B**) Rarity Index, (**C**) Observed Richness, and (**D**) Shannon Diversity. Box boundaries are the interquartile range (IQR); whiskers 1.5 times the IQR; mid-line inside the box is the median. Data from the three stations are combined because PERMANOVA showed no significant differences among stations for any of these indices. Significant differences among different depths are indicated by different lower-case letters

The Dominance Index (DI) was based on the abundance of 170 core ASVs (Supplementary File 1, Table S2) and varied significantly across depths (*p* = 0.004), but not stations (Fig. 4A; Table 1). The DI for the D5 assemblage (0.160 ± 0.01) was lower than the DI for the assemblages at D1 (0.42 ± 0.06, pairwise PERMANOVA: *p* = 0.001) and at D2 (0.40 ± 0.06, *p* = 0.002). Similarly, the Rarity Index (RI), which refers to the relative proportion of the non-core species, varied significantly across depths (*p* = 0.020) but not stations (Fig. 4B; Table 1). The D5 assemblage RI was 0.68 ± 0.01, which was higher than the RI for assemblages at D1 (0.39 ± 0.04, *p* = 0.014) and D2 (0.41 ± 0.06, pairwise PERMANOVA: *p* = 0.020). Observed dinoflagellate ASV richness ranged from 165 to 1,027 ASVs per sample (Fig. 4C), but there was no significant difference in observed richness among depths or stations (Table 1). The Shannon Diversity index ranged from 3.67 to 5.64 per sample, and like richness, there was no significant difference in Shannon Diversity among depths or stations (Fig. 4D; Table 1).

### Conventional morphological analysis

Conventional morphological analysis of taxa in seawater samples collected at Station C identified 52 phytoplankton taxa, of which 17 were dinoflagellates in class Dinophyceae (Table 2, Supplementary File 1, Table S5). In comparison, amplicon sequencing of eDNA in seawater samples collected at Station C recovered 2,065 ASVs representing 4 classes, 13 orders, 65 families, 33 genera, and 39 species (at each taxonomic level there were unclassified ASVs). The number of taxa identified by metabarcoding but not by morphology was 38 at the level of species and 30 at the level of genus. Thirteen of the taxa identified by morphological analysis were not identified by metabarcoding (Table 2), and in all but six of these cases, equivalent taxonomic rank information was absent in the PR2 database.

**Table 2:** Abundance (cells/ml) of dinoflagellate taxa detected by microscopy methods throughout the water column to a depth of 150 m beneath the sea surface (D1: 5–10 m, D2: 20–30 m, D3: 45–60 m, D4: 95–120 m, D5: 130–150 m) at Station C. Taxa in bold were not identified by metabarcoding. Asterisks indicates taxa entry absent from PR2 database. Hyphen indicates taxa not detected. x denotes species detected in sample but not observed during cell counts

Taxa	Depth				
	D1	D2	D3	D4	D5
Blepharocysta splendormaris*	-	25	-	-	-
Citharistes regius*	-	25	-	-	-
*Margalefidinium *spp.	-	50	25	200	-
Dinophysis schuetii*	-	-	50	-	-
**Diplopsalidaceae**	-	-	-	25	-
***Gonyaulax ***spp.	-	100	25	50	-
*Gymnodinioid *spp.	2,400	2,200	3,500	2,400	4,000
*Gyrodinium *spp.	700	1,100	800	500	1,000
*Heterocapsa rotundata*	500	1,200	700	400	200
Katodinium glaucum*	200	500	100	25	200
Mesoporos perforatus*	200	200	-	-	-
***Oxytoxum ***spp.*	1,500	1,600	1,000	800	-
***Peridinium ***sp.	100	200	200	-	-
***Phalacroma rotundatum***	-	25	-	-	-
***Protoperidinium ***spp.	100	25	-	50	-
***Scrippsiella ***spp.	x	-	50	25	200
***Thoracosphaera heimii***	-	-	-	-	200

Identification of four dinoflagellate taxa by morphology was consistent with taxa identified by metabarcoding methods (shown in non-bold text in Table 2). Of these, one was identified at the species level (*Heterocapsa rotundata*, which was represented by 16 ASVs), whereas the remaining taxa were identified at the family or genus level. For the latter, metabarcoding could allocate or differentiate species and/or species variants (ASVs are species/subvariant analogs). Specifically, metabarcoding identified *Margalefidinium* spp. as *Margalefidinium polykrikoides* (2 ASVs), *Gymnodinioid spp.* as 269 ASVs in family Gymnodiniales of which 17 were allocated species-level taxonomy, and *Gyrodinium spp.* as 52 ASVs which included *Gyrodinium gutrula, Gyrodinium heterogrammum, Gyrodinium fusiform, Gyrodinium spirale, Gyrodinium dominans, Gyrodinium helveticum*, and *Gyrodinium rubrum*.

### Environmental parameters

**Fig. 5:**
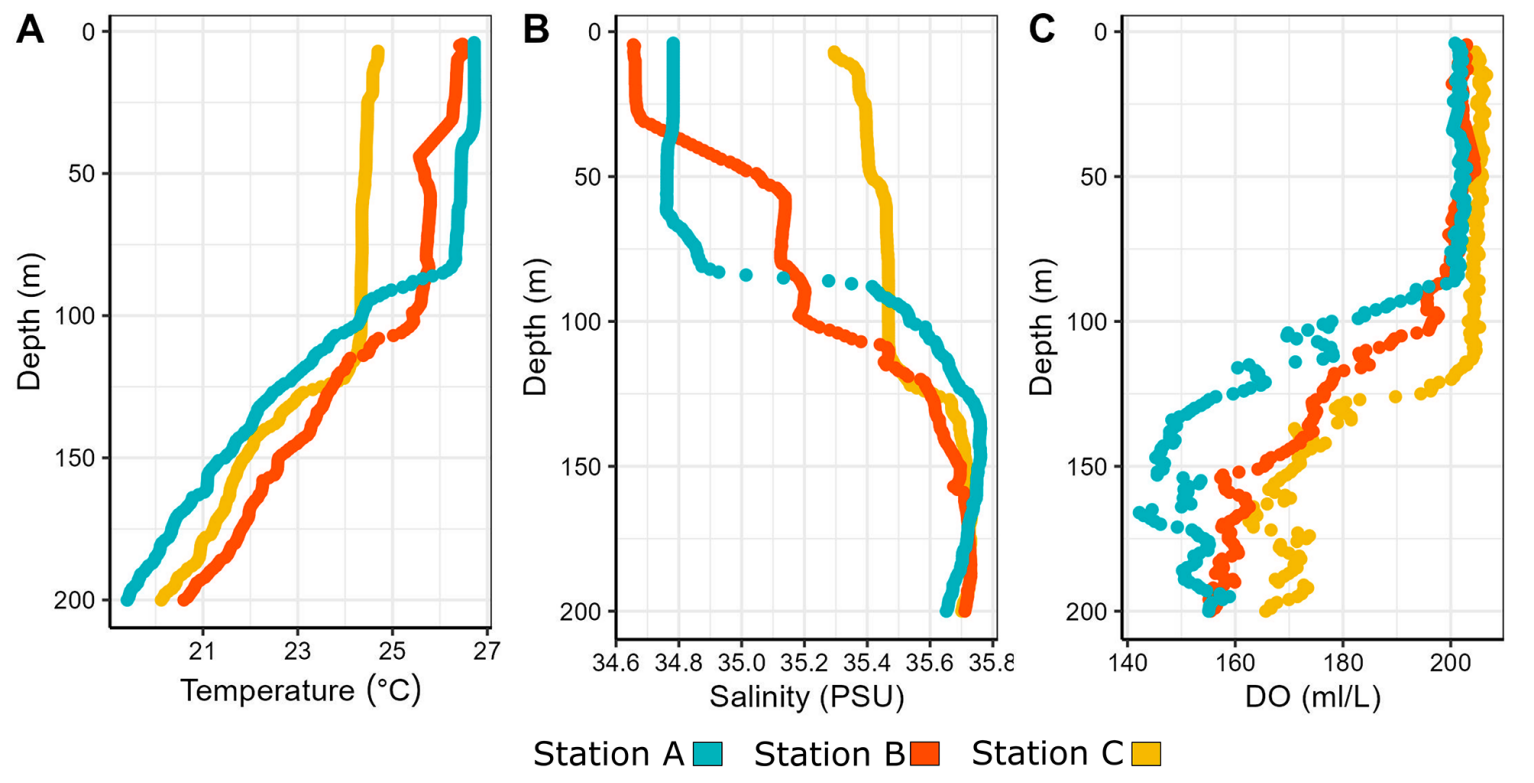
Conductivity-temperature-depth (CTD) profile data for each station: (**A**) temperature, (**B**) salinity, and (**C**) dissolved oxygen (DO). Colours indicate stations: Station A is blue, Station B is orange, and Station C is yellow. Data from 0 to 200 m below sea surface

At the three stations, the conductivity-temperature-depth (CTD) profiles showed stratification of the water column with clines in temperature and salinity at 40–125 m below the surface (Fig. 5A&B). PAR minima were found around 40 m at Station A and C, and around 60 m at Station B (Supplementary File 1, Figure S2B). Dissolved oxygen began to decline at 100 m at Stations A and C, and at 125 m at Station B (Fig. 5C), and at all three stations oxygen minima occurred around 150 m. Turbidity at all three stations peaked between 100 and 130 m (Supplementary File 1, Figure S2A). A summary of environmental parameters estimated from CTD casts for each station at each depth is provided in Supplementary File 1, Table S6.

## Discussion

In this study, we assessed the diversity and vertical distribution of dinoflagellate assemblages throughout the water column to a depth of 150 m beneath the sea surface at three stations in open-ocean water masses in the Western Coral Sea, Australia. Using metabarcoding of the V4 region of 18 S rDNA, a total of 5,103 dinoflagellate ASVs were recovered. Taxonomy annotated with the PR2 database identified dinoflagellate taxa represented by 4 classes, 14 orders, 76 families, 51 genera, and 63 species. Conventional morphological analysis complemented this approach by identifying 13 additional dinoflagellate taxa. The composition of major dinoflagellate groups displayed varying vertical and latitudinal distribution patterns. Interestingly, despite stations being separated by hundreds of kilometres, the influence of latitudinal gradient on dinoflagellate assemblages, while significant, explained less of the variation in dinoflagellate assemblage structure than depth. Previously, the Coral Sea was shown to be a biodiversity hotspot for metazoan and micronekton taxa [26–28, 30, 31]. To our knowledge, our study is the first to demonstrate that this diversity extends to dinoflagellates in open-ocean water masses of the Western Coral Sea. The findings of this study have implications for food web structure, energy flow and biogeochemical cycling in this marine ecosystem.

### The vertical distribution of dinoflagellates

The two main dinoflagellate classes, Dinophyceae (phototrophic and heterotrophic) and Syndiniales (parasitic) were consistently abundant based on their ASV representation across all stations and depths. These two classes, however, displayed contrasting patterns of abundance and distribution along the depth gradient that reflected differences in nutritional strategies. Dinophyceae were a significant proportion of the upper euphotic zone samples, and their relative abundance decreased gradually with depth. The upper epipelagic zone is where photosynthetically active radiation is highest during daylight hours. In contrast, Syndinians’ relative abundances increased with depth and were particularly abundant in the lower euphotic zone, which is nutrient and prey resource rich. Taxonomically, Syndiniales are divided into groups I–V [38], and despite being widespread in the open ocean, they are particularly abundant in oxygen-depleted water columns [39–41]. In our study, all four Syndiniales groups were most abundant at 130–150 m (D5), which coincided with the oxygen minima observed across the three stations. Indeed, Syndiniales Group-I, the most abundant Syndiniales group overall, was almost five-fold more abundant at 130–150 m (D5) than in the upper epipelagic zone. This is consistent with previous reports showing Syndiniales is associated with low oxygen and euphotic ecosystems in other regions including the Caribbean and Mediterranean Seas [38, 41], and the Atlantic and Eastern Tropical North Pacific Oceans [39, 41].

The varying abundance and distribution patterns observed for some taxonomic and functional groups among depths reflected differing environmental conditions created by prevailing currents, light gradients, resource availability, and physical and chemical properties of the water column [1, 42, 43]. Assemblages at 130–150 m (D5) were within the typical boundaries of Subtropical Lower water, which presents different environmental conditions than at shallower depths [42, 43]. At all stations, assemblages at 5–30 m (D1 and D2) were highly similar, and were within the mixed layer depth typically observed in this region during austral winter, thus subject to wind-induced vertical mixing [44]. During daylight, photosynthetically active radiation is highest in the upper epipelagic zone, enabling photosynthesis; in contrast, the lower euphotic zone is nutrient-rich with high inorganic and prey resources. Assemblage structure varied the greatest among all stations at 95–150 m (D4 and D5). Stations were located at different latitudes and within different major current systems, characterized by various physical and chemical water parameters in the upper water column [45]. However, overall, the influence of spatial variability on the relative abundance of dinoflagellate ASVs, while important, was less influential than depth. This reinforces that dinoflagellate taxa aggregate within an optimal depth range for survival based on adaptation to prevailing environmental conditions and prey and resource availability, and that dinoflagellate assemblages are similar in biogeographic zone A as described previously [1, 3, 10].

Transitions in assemblage composition were observed between sampled depth ranges and may be explained by a combination of vertical migration via flagellar motility and diffusion and circulation caused by currents, tidal mixing, and upwelling [41, 46, 47]. Swimming speeds of dinoflagellates are ≤ 60 m per day; thus, flagellar motility may explain some of the similarities observed in assemblages at 5–60 m (D1– D3) but does not explain distribution patterns spanning larger distances observed for some taxa. In these cases, deeper assemblages may have been seeded from surface mixotrophic populations that survived at depth by relying more on heterotrophic metabolism [1]. Diffusion and circulation of free-living dinoflagellate spores, such as Syndiniales spores, contributes to distribution patterns spanning large distances [41].

The vertical stratification pattern observed for the major dinoflagellate taxonomic groups in our study is consistent with previous reports on the vertical partitioning of marine protists in the water column [1, 10, 40, 41, 48]. Ollison et al. [10], for example, found protist communities partitioned into three distinct assemblages along the depth gradient, with significant changes occurring between 75 and 100 m and 175–300 m. Schnetzer et al. [48] found that assemblages in the lower water column (≥ 150 m) were distinct from shallower depths. Similarly, we found assemblages at 130–150 m (D5) were highly similar to each other across stations but were distinct from assemblages at shallower depths.

### The distribution of HAB forming taxa

There were important differences in the relative abundances of HAB-forming species among stations. In addition, the abundance of non-HAB forming *Tripos* spp., which has been suggested as a key genus for defining climate-based changes to the world’s oceans [49], increased with decreasing latitude. Overall, the abundance of HAB species was greatest at Station A. The HAB forming *Karenia brevis* was detected at all three stations, but was over three-fold more abundant at Station A than at Stations B and C. Although detected at low relative abundances ∼ 0.04%), this is only the second report of this HAB species in Australian waters [13]. Previously, *K. brevis* was considered restricted to waters of the Gulf of Mexico, however, it is important to note the possibility that this was a ASV misassignment. It has been noted that the species *K. brevis, K. mikimotoi* and *K. papilionacea* shared identical V4 reference sequences [19]. Here, the reference sequences for *K. brevis* and *K. mikimotoi* shared 99.77% sequence identity, differing by just four nucleotides, thus *K. brevis* identified in Australian waters may be the similar species *K. mikomotoi*. To confirm the presence of *K. brevis*, further sampling with both traditional and molecular methods is needed. Other HAB species, such as *H. neirotundata*, *L. chlorophoru*, and *M. fulvescens* and *K. veneficum* [49, 50], were also detected in this study, highlighting the usefulness of eDNA metabarcoding for monitoring toxic/HAB species with potential to impact both human health and commercial fisheries. The geographic range of some HAB taxa, and the frequency of HAB events, is increasing due to climate change and anthropogenic impacts (e.g. marine pollution and eutrophication) [51, 52]. In addition to monitoring HAB distribution, eDNA metabarcoding has potential for assisting in monitoring climate-based changes in the world’s oceans.

### Challenges and opportunities in assessing dinoflagellate assemblages using molecular methods

Metabarcoding of eDNA has furthered our understanding of the species assemblages and spatial distribution patterns of marine protists. Molecular methods have made it possible to characterize new diversity, revealing rare and cryptic taxa, that would have been challenging with traditional microscopy and culture-dependent methods [53, 54]. ASV richness for free-living dinoflagellate species in this study was higher than the number of currently described free living species [55]. This alludes to a high number of dinoflagellate-crypto and -morphospecies in the environmental samples; however, it also alludes to the potential influence of intragenomic diversity on estimates of assemblage diversity and richness. In this regard, the DADA2 pipeline is particularly adept at chimera removal and distinguishing true biological variation from sequencing artifacts [54, 56]. Further, ASV-based methods, compared to OTU-methods, are more able to capture intragenomic diversity and provide a more detailed representation of the diversity present. Biological and technical biases, however, can lead to an overestimation of ASV abundance, and an inflation of richness and diversity indices [57, 58]; consequently, it is necessary to interpret results cautiously. This challenge is particularly pertinent for studies involving dinoflagellates, which compared to other protists, are known for their large genome size and high rRNA gene copy number, with intragenomic diversity and genomic copy number varying among species [57, 59–61]. Thus, even relative abundance of taxa, which is often accepted as being proportionately representative of the community, has limitations when applied in assessments of dinoflagellate diversity.

In our study, the number of unclassified ASVs at the family-species level was high, with only 63 species identified, of which many were HAB forming species, and one was a Syndinian. It is widely accepted that taxonomic reference databases are biased towards taxa that can be cultured under laboratory conditions and taxa of interest, which in the case of dinoflagellates are mainly toxic and HAB-forming species [10, 62]. In contrast, less effort has gone into characterizing other dinoflagellate functional groups (e.g. parasitic species) and species found in open ocean areas [23]. The PR2 database v.4.14 incorporates DINOREF (a curated dinoflagellate database) and contains 15,772 reference sequences for 573 dinoflagellate species. Class Dinophyceae is the largest contributor to reference species in PR2 (490 species), accounting for 6,720 reference sequences. In comparison, Syndiniales is only represented by 72 species and accounts for 8,977 reference sequences. One approach to improve taxonomic resolution is to combine molecular approaches with morphological analysis [63, 64]. Accordingly, we found conventional morphological analysis complemented eDNA metabarcoding by increasing the number of dinoflagellate taxa identified.

The limited species-level data in taxonomic reference databases is a substantial drawback to the many advantages provided by metabarcoding [1, 23]. Taxonomic assignment of ASVs, particularly at lower taxonomic levels, is necessary for studying ecological significance. For instance, all described Syndiniales are parasitoids and can have specific or non-specific host associations. Hosts are other protists (radiolarians, dinoflagellates) or metazoans (copepods, crabs, fish eggs) that are killed upon dinospore release [46]. Syndiniales group II taxa, for example, have important roles in controlling HABs by the mechanism of parasitism [65]; consequently, their abundance and distribution can have major ecological, economic, and societal impacts [36, 37]. Parasitism of Syndiniales on plankton can influence plankton population dynamics, thus having important implications for ocean food webs and biogeochemical cycling which are connected via trophic interactions [66–69]. The number of unclassified ASVs at the family-species level was particularly high for Syndinian ASVs with only one Syndiniales species identified, *Syndinium turbo*, a species that parasitizes copepods [66]. Future research should focus on characterizing Syndinians with the aim of increasing their representation in taxonomic reference databases, and to improve our understanding of their role in marine ecosystems.

## Conclusion

This study provides detailed insight into the diversity of dinoflagellate assemblages in the Western Coral Sea, Australia, which to our knowledge, has not been rigorously addressed in previous ecological studies. Dinoflagellate assemblages were diverse, and taxonomic groups differed in their vertical distribution throughout the water column to a depth of 150 m yet were similar across the horizontal scale of our study. Vertical distribution patterns reflected differences in environmental conditions related to major ocean currents in the Coral Sea. Considerable unknown diversity was discovered, highlighting gaps in knowledge regarding taxonomic characterization and representation of dinoflagellates in existing databases. The identification of HAB dinoflagellates in the study reinforces that metabarcoding is a useful tool for monitoring HABs. Overall, this study is an important step in improving our understanding of dinoflagellate diversity in the Coral Sea and may improve our understanding of plankton community dynamics, and it may assist in refining ecosystem models that can help monitor and predict environmental change.

## Methods

**Fig. 6:**
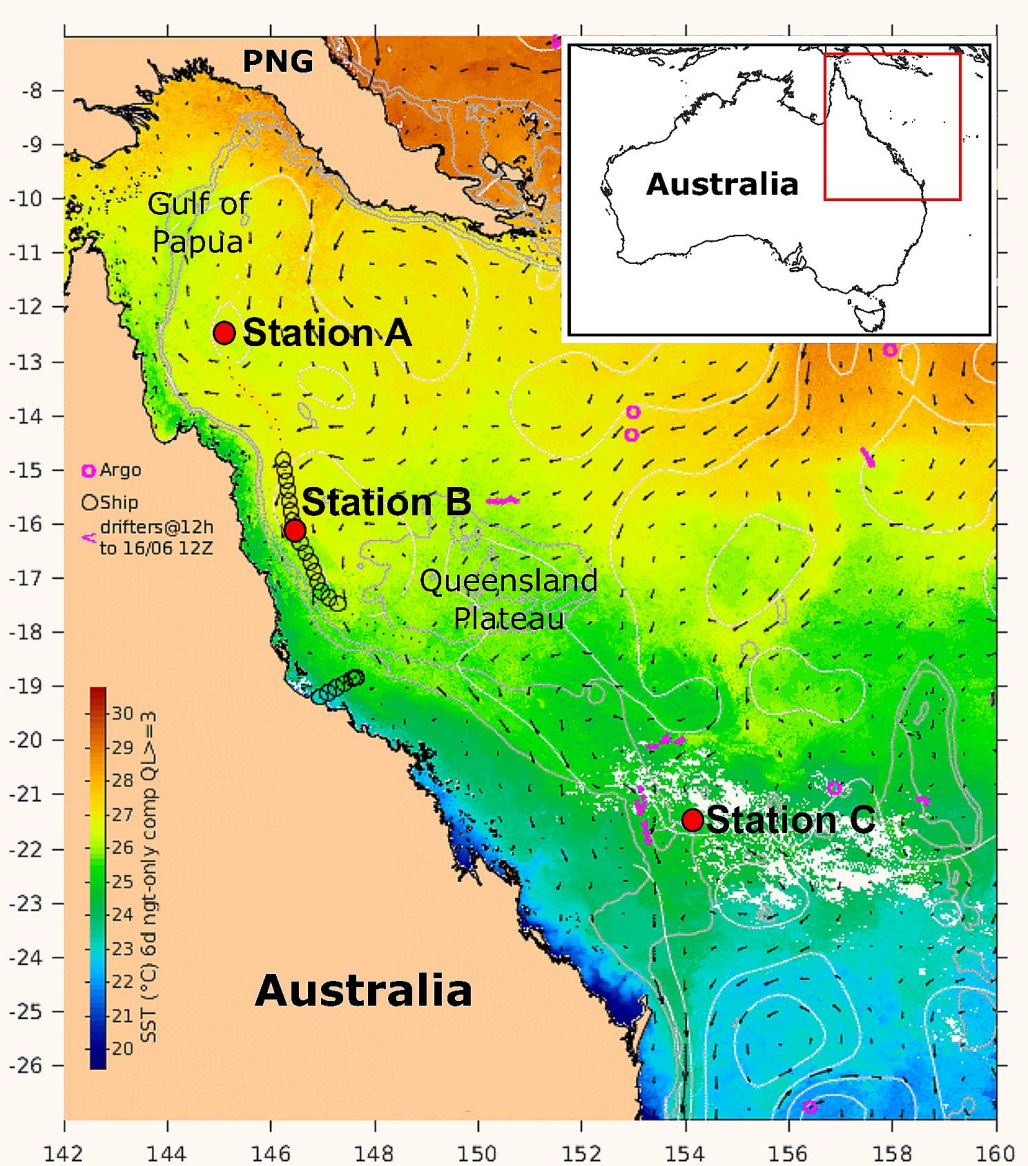
Map of sea surface temperatures (SSTs) showing currents (denoted by black arrows) and stations (Station A, B and C) where seawater samples were collected from depth zones (D1: 5–10 m, D2: 20–30 m, D3: 45–60 m, D4: 95–120 m, D5: 130–150 m) during a transect through the Western Coral Sea in June 2021 (austral winter). Colours are a mean composite of remotely sensed SST in a 6-day window centered on June 15, 2021 (https://oceancurrent.aodn.org.au/). Depth contours are marked with white lines **Additional files**

Samples were collected at three stations (Station A, B, and C) during a transect through the Coral Sea in June 2021 (austral winter) (Fig. 6). Maximum depth at the stations exceeded 1,000 m. Stations A, B, C were sampled on June 16, 15, and 12, respectively. Stations A and B were located near the edge of the Great Barrier Reef and the Queensland Trough and Station C was located near the Marion Plateau and Cato Trough.

In the study region, surface water is characterized by temperatures > 24 °C and salinity between 34.5 and 35.5 ppt [42, 43]. The circulation of surface water is influenced by three major currents, the South Equatorial Current (SEC), Hiri Current (HC), and the East Australian Current (EAC). The SEC, which is on average, about 150 m thick, flows from the east and bifurcates on the GBR into a northern arm (the North Queensland Current) and a southern arm (EAC) [45]. The location of the bifurcation from December to March is around 14 °S, and then it moves toward 20 °S April to November. The depth of the mixed layer, the surface water in which salinity and temperature are vertically quasi-homogeneous, varies spatially and seasonally [44]. The mixed layer depth (MLD) is shallower in the austral summer than in winter. Typically, in winter tropical MLDs range from 50 to 100 m, whereas in the south-western Coral Sea winter MLDs often exceed 100 m and reach up to 240 m [44]. Beneath the surface water lies Subtropical Lower water (SLW), which is characterized by a temperature of 18 to 25 °C, salinity of 35.5 to 36.0 ppt and depth of 50 to 150 m. Beneath the SLW lies Antarctic Intermediate water, which is characterized by a temperature of 4.2 to 9.0 °C, salinity of 34.4–34.8 ppt and depth of 500 to 1,200 m [42, 43].

### Sample processing

At each station, seawater (15 L) was collected from five depth zones: depth 1 (D1, 5–10 m), depth 2 (D2, 20–30 m) depth 3 (D3, 45–60 m) depth 4 (D4, 95–120 m) and depth 5 (D5, 130–150 m) at night-time using Niskin bottles attached to a rosette sampler. Samples were filtered through a 50 μm mesh and 4 L was allocated for eDNA isolation, and an additional 1 L was allocated from each depth at Station C for morphological identification. Prior to analysis, samples for eDNA isolation were filtered onto 5.0 μm pore size self-preserving (SP) eDNA filter packs (Smith-Root), sealed in the supplied storage bags, and kept in darkness at room temperature. Samples for morphological identification were preserved with Lugol’s iodine solution (0.1–0.5% final concentration) and stored in insulated containers in darkness at room temperature. Morphological identification was undertaken by Microalgal Services, Ormond, Victoria, Australia.

At each site, water profile data were collected during the daytime using a CTD logger (SeaBird) attached to a rosette sampler. Environmental measurements included pressure, temperature, salinity, oxygen (SBE43 Oxygen, Sea-Bird Scientific), fluorescence (WET Labs ECO-AFL/FL), turbidity (WET Labs ECO BB), photosynthetically active radiation (PAR) and transmittance (Wetlabs C-Star).

### DNA isolation, amplification, and sequencing

eDNA was isolated from SP filters using a modified CTAB protocol [13]. All eDNA isolations were undertaken in a dedicated, sterilized DNA-only extraction hood. Any inhibitors that may have been present in seawater samples were removed by application to One Step PCR inhibitor removal columns (Zymo) following the manufacturer’s instructions. The quantity and purity of template DNA were assessed using a Nanodrop spectrophotometer (PicoDrop Ltd, Hinxton, UK). PCRs were undertaken using 20 ng of eDNA under the conditions and with the primer and adaptor sequences shown in Table 3. All unpooled sample amplicons were sequenced by the Ramiciotti Centre for Genomics (University of New South Wales) using paired-end Illumina sequencing on the MiSeq platform.

**Table 3:** Sequences of primer pairs with Nextera Illumina adaptors. Target, product size, and reaction conditions for real-time PCR assays were the same for both primers

Primer and adaptor sequences	Target	Product size	Thermocycling conditions
Forward Primer V418SNextFor:	18 S rRNA V4	378 bp	5 min at 95 °C, 30 × (30 s at 95 °C, 30 s at 55.2 °C, 30 s at 72 °C), 5 min at 72 °C.
5’-[TCGTCGGCAGCGTCAGATGTGTATAAGAGACAG] CCAGCASCYGCGGTAATTCC-3’			
Reverse primer V418SNextRev:			
5’-[GTCTCGTGGGCTCGGAGATGTGTATAAGAGACAG] ACTTTCGTTCTTGATYRATGA-3’			

### Bioinformatics and statistical analyses

All bioinformatic and statistical analyses were performed using RStudio (v.2022.07.1, R v.4.1.3). Demultiplexed samples were filtered, trimmed, dereplicated, and denoised paired reads were merged, and chimeras removed using the DADA2 pipeline v.1.16.0 with default parameters [54]. Forward and reverse trim parameters were set as truncLen = c(280, 200), trimLeft = c(20, 21). Amplicon Sequence Variants (ASVs), also known as zero radius OTUs, were assigned using the assignTaxonomy algorithm in DADA2 with default values against the PR2 database v.4.14.0 [70]. Tables produced by DADA2 were converted into a phyloseq object using the R package phyloseq v.1.38.0 [71]. The ASV matrix was filtered to remove ASVs not assigned to a division and ambiguously assigned ASVs (multiple species assignment). ASVs that contained one sequence within the entire data set were removed. Then the ASV matrix was subset by Division Dinoflagellata, which was the most abundant group across all samples (Table S7, Supplementary File 1).

For compositional plots and downstream analysis, raw ASV sequence counts were normalized to the median sequencing depth using transform_sample_counts(data, function(x, t = median(sample_sums(data))) round(t*(x/sum(x)))) in the phyloseq. Supplementary File 2 contains a table of normalized abundances for ASVs at each depth at each station. Ordination using the Bray-Curtis distance was generated by applying Principal Coordinates Analysis (PCoA) to Hellinger-transformed normalized abundance data using *transform(“hellinger”)* in the microbiome R package [72], and *ordinate(“PCoA”)* in phyloseq. Heatmap graphic was created using ordination methods [73], implemented with the function *plot_heatmap(“RDA”)* in the phyloseq R package. To investigate the influence of depth and station location, permutational multivariate analyses of variance (PERMANOVA) was conducted using the function *adonis.2(nperm = 999)* in the vegan R package [74]. Post hoc tests for significant PERMANOVAs were pairwise PERMANOVAs (ADONIS) with P values adjusted using Holm’s method [75] and were performed using the function *pairwise.adonis()* in the pairwiseAdonis R package [76]. Indicator analysis was used to identify ASVs most characteristic of a depth or station. Normalized abundance data were transformed to presence-absence, and the function *multipatt(func="r.g”, (nperm = 999))* in the R package Indicspecies was used to calculate the phi (ϕ) correlation coefficient and the strength of association for each ASV to a group (depth or station) or combination of groups [77].

Alpha diversity indices (observed richness, Shannon index, core abundance dominance index, and rare (non-core) abundance rarity index) were calculated from raw read count data using the microbiome R package [72]. Observed richness is the number of ASVs detected in a sample. Shannon index is calculated as:

$$ {H}^{{\prime }} = - \sum _{i=1}^{S}{p}_{i}{ln}_{b}{p}_{i}$$

where $$ {p}_{i}$$ is the proportional abundance of ASV $$ i $$, and $$ S $$is the number of ASVs so that $$ {\sum }_{i=1}^{S}{p}_{i} = 1$$, and $$ b $$is the base algorithm [74]. Core abundance refers to the relative proportion of the core ASVs, defined as ASVs with over 50% prevalence at 0.2% relative abundance. Rare abundance refers to the relative proportion of least abundant taxa (non-core taxa) within each sample, regardless of the population prevalence. For the ASVs assigned a binomial species name, the HAB forming species were identified using information available in the literature [23, 50, 78, 79]. Statistical significance was set at *P* < 0.05. Unless otherwise stated, data are mean ± standard deviation, and relative abundance of dinoflagellate taxa is expressed as a percentage of the total number of dinoflagellate normalised reads (2,382,290 reads).

### Electronic supplementary material

Below is the link to the electronic supplementary material.

Supplementary File 1: Binomial names of the 63 identified species and if they are a known harmful algal bloom (HAB) forming species; Taxonomy and raw read abundance of the ASVs classified as core; Indicator ASVs for stations and depths; Morphological identifications; Environmental data; Division summary; Rarefaction curves; CTD cast data.

Supplementary File 2: Normalized abundances of Dinoflagellata ASVs.

Supplementary File 3: Summary of dinoflagellate taxa.

## Data Availability

The datasets supporting the conclusions of this article are included within the article and its supplementary files.
