# Supplementary material for: eDNA metabarcoding reveals biodiversity and depth stratification patterns of dinoflagellate assemblages within the epipelagic zone of the western Coral Sea
Source: BMC Ecol Evol. 2024 Mar 26;24:38. doi: 10.1186/s12862-024-02220-7 (PMC10964515; doi:10.1186/s12862-024-02220-7)
Supplement: Supplementary file 3 — Summary of dinoflagellate taxa. [file 12862_2024_2220_MOESM3_ESM.docx]

# Supplementary file 3

## Class: Dinophyceae, order Gymnodiniales

Within the order Gymnodiniales, family Gymnodiniaceae, ASVs were affiliated with 10 genera, but mainly represented by the genera *Gyrodinium* (130,630 reads, 91 ASVs) and *Gymnodinium* (130,560 reads, 112 ASVs). *Gyrodinium* was represented by *Gyrodinium fusiforme* (37,326 reads, 23 ASVs), *Gyrodinium dominans* (31,793 reads, 18 ASVs), *Gyrodinium helveticum* (18,002 reads, 7 ASVs), *Gyrodinium spirale* (8,445 reads, 4 ASVs), *Gyrodinium heterogrammum* (1,801 reads, 2 ASVs), *Gyrodinium rubrum* (308 reads, 1 ASV), *Gyrodinium gutrula* (273 reads, 6 ASVs), and *Gyrodinium* spp. (32,682 reads, 30 ASVs). *Gymnodinium* was represented by *Gymnodinium dorsalisulcum* (8,525 reads, 10 ASVs), *Gymnodinium aureolum* (5 reads, 1 ASV) and *Gymnodinium* spp. (122,030 reads, 99 ASVs).

The less abundant Gymnodiniaceae genera were *Lepidodinium* represented by *Lepidodinium chlorophorum* (17,535 reads, 12 ASVs), *Lepidodinium viride* (28 reads, 1 ASV), and *Lepidodinium* spp. (4,354 reads, 26 ASVs), followed by *Margalefidinium*, which was represented by *Margalefidinium polykrikoides* (3,836 reads, 15 ASVs) and *Margalefidinium fulvescens* (2,617 reads, 13 ASVs), *Gymnoxanthella* (482 reads, 7 ASVs classified as *Gymnoxanthella radiolariae*), *Amphidinium* which was represented by *Amphidinium* sp. (320 reads, 3 ASVs) and *Amphidinium longum* (36 reads, 1 ASV), *Polykrikos* (88 reads, 4 ASVs classified as *Polykrikos* sp./spp.), *Paragymnodinium* (81 reads, 4 ASVs classified as *Paragymnodinium shiwhaense*), *Balechina* (80 reads, 1 ASV classified as *Balechina pachydermata*), *Ankistrodinium* (6 reads, 1 ASV classified as *Ankistrodinium semilunatum*).

The other Gymnodiniales families were Warnowiaceae (112,256 reads, 121 ASVs), which was represented by *Warnowia* sp. (99,897 reads, 93 ASVs) and *Erythropsidinium agile* (92 reads, 6 ASVs), Kareniaceae (28,545 reads, 52 ASVs) which was represented by *Karlodinium veneficum* (11,432 reads, 15 ASVs), *Karlodinium* spp. (14,687 reads, 17 ASVs), *Karenia brevis* (1,026 reads, 4 ASVs), *Karenia mikimotoi* (95 reads. 1 ASV), and *Karenia* sp./spp. (584 reads, 8 ASVs), Chytriodiniaceae (10,877 reads, 18 ASVs) which was represented by *Chytriodinium roseum* (7439 reads, 7 ASVs), *Chytriodinium affine* (2,170 reads, 4 ASVs), *Chytriodinium* spp. (1,023 reads, 6 ASVs), and Ceratoperidiniaceae (4,300 reads, 11 ASVs classifed as *Ceratoperidinium falcatum*).

## Class: Dinophyceae, order Prorocentrales

All ASVs affiliated with Dinophyceae, order Prorocentrales (124,018 reads, 155 ASVs) were in the family Prorocentraceae, with no further taxonomy allocated.

## Class: Dinophyceae, order Peridiniales

The Dinophyceae order Peridiniales (119,693 reads, 280 ASVs) was represented by seven families, of which Heterocapsaceae (40,075 reads, 22 ASVs) and Blastodiniaceae (33,343 reads, 58 ASVs) were at least three-fold more abundant than the other families. Heterocapsaceae was mainly represented by *Heterocapsa nei/rotundata* (37,886 reads, 12 ASVs), but also included ASVs affiliated with *Heterocapsa pygmaea* (40 reads, 1 ASV), and *Heterocapsa* sp./spp. (2,149 reads, 9 ASVs). Blastodiniaceae were represented by *Blastodinium mangini* (25,565 reads, 30 ASVs), *Blastodinium contortum* (6,616 reads, 12 ASVs), *Blastodinium crassum* (480 reads, 6 ASVs), *Blastodinium spinulosum* (224 reads, 2 ASVs), *Blastodinium galatheanum* (63 reads, 2ASVs), and *Blastodinium* spp. (395 reads, 6 ASVs).

The less abundant Peridiniales families included Thoracosphaeraceae (10,110 reads, 39 ASVs), Amphidomataceae (2,428 reads, 14 ASVs), Podolampadaceae (1,239 reads, 13 ASVs), Hetrodiniaceae (813 reads, 2 ASVs, only *Heterodinium milneri*), and Protoperidiniaceae (147 reads, 4 ASVs). Thoracosphaeraceae was represented by *Luciella* sp. (78 reads, 1 ASV), *Pentapharsodinium* spp. (2663 reads, 6 ASVs), *Pentapharsodinium tyrrhenicum* (881, 9 ASVs), *Scrippsiella acuminata* (499 reads, 2 ASVs), *Scrippsiella* sp. (66 reads, 1 ASV), *Thoracosphaera heimii* (17 reads, 1 ASV), *Thoracosphaera* spp. (5,906, 19 ASVs). Amphidomataceae was represented by *Azadinium trinitatum* (1,060 reads, 2 ASVs), *Amphidoma languida* (72 reads, 1 ASV), and *Azadinium* spp. (1,296 reads, 11 ASVs). Podolampadaceae was represented by *Lessardia elongata* (1,220 11 ASVs) and *Podolampas* sp./spp. (19 reads, 2 ASVs). Protoperidiniaceae was represented by *Protoperidinium bipes* and *Protoperidinium* sp. *(*31 reads, 2 ASVs).

## Class: Dinophyceae, order Gonyaulacales

The Dinophyceae order Gonyaulacales (11,966 reads, 43 ASVs) was represented by the families Ceratiaceae, Gonyaulacaceae, Goniodomataceae, and Pyrocystaceae. Ceratiaceae ASVs (10,285 reads, 27 ASVs) were affiliated with *Tripos gravidus* (4,702 reads, 5 ASVs)*, Tripos furca* (3,877 reads, 8 ASVs)*, Tripos fusus* (1,131 reads, 4 ASVs)*, Tripos digitatus* (303 reads, 4 ASVs)*, Tripos contrarius* (208 reads, 3 ASVs), *Tripos* sp./spp. (64 reads, 3 ASVs). Gonyaulacaceae (862 reads, 5 ASVs) was represented by *Lingulodinium polyedra* (815 reads, 3 ASVs) and *Gonyaulax* sp. (47 reads, 2 ASVs). Goniodomataceae (556 reads, 8 ASVs) was mainly represented by *Goniodoma polyedricum* (388 reads, 3 ASVs), and to a lesser extent by *Alexandrium cohorticula* (56 reads, 1 ASV)*, Alexandrium hiranoi* (48 reads, 1 ASV)*, Alexandrium tamiyavanichii* (40 reads, 1 ASV)*, Alexandrium pohangense* (9 reads, 1 ASV)*,* and *Alexandrium* sp. (15 reads, 1 ASV). Pyrocystaceae (263 reads, 3 ASVs) was represented by *Pyrocystis pseudonoctiluca* (149 reads, 2 ASVs) and *Pyrocystis* sp. (114 reads, 1 ASV).

## Class: Dinophyceae, order Torodiniales

The Dinophyceae order Torodiniales (8,493 reads, 53 ASVs) was affiliated with the family Torodiniaceae (8,493 reads, 53 ASVs), and was mainly represented by *Torodinium robustum* (8,100 reads, 34 ASVs); whereas rarely observed were *Torodinium teredo* (184 reads, 7 ASVs), and *Torodinium* spp. (209 reads, 12 ASVs).

## Class: Dinophyceae, order Suessiales

The Dinophyceae order Suessiales (5,600 reads, 18 ASVs) was represented by ASVs affiliated with the family Suessiaceae (5,559 reads, 17 ASVs), which were affiliated to the genera *Pelagodinium* (*Pelagodinium beii*, 2,952 reads, 8 ASVs, and *Pelagodinium* sp., 1,195 reads, 2 ASVs), *Protodinium* (*Protodinium simplex*, 830 reads, 4 ASVs), and *Biecheleria* (*Biecheleria sp.*, 537, 2 ASVs). The Suessiales family Symbiodiniaceae was represented *Symbiodinium* sp. (41 reads, 1 ASV).

## Class: Dinophyceae, order Dinophysiales

Rarely observed was the Dinophyceae order Dinophysiales (100 reads, 2 ASVs), represented by *Phalachroma* sp. (54 reads, 1 ASV) and one ASV in the family Dinophysaceae (Dinophysaceae_X_sp., 46 reads).

## Class: Syndiniales

## Class: Syndiniales, Dino-Group-III

Representatives of Syndiniales Dino-Group-III (32,280 reads, 140 ASVs) were about three-fold more abundant than Dino-Group-IV (9,845 reads, 23 ASVs). All Dino-Group-III ASVs were of an undetermined family/group (Dino-Group-III_X).

## Class: Syndiniales, Dino-Group- IV

Syndiniales Group-IV ASVs were affiliated with groups Dino-Group-IV-Hematodinium-Group (5,557 reads, 14 ASVs) and Dino-Group-IV-Syndinium-Group (4,288 reads, 9 ASVs). Syndiniales of an undetermined order/group (Syndiniales_X) accounted for 433 reads (3 ASVs). Only one Syndiniales species was identified, *Syndinium turbo*, Dino-Group-IV-Syndinium-Group (2,777 reads, 3 ASVs).

## Class: Syndiniales, Dino-Group- V

Syndiniales Dino-Group-V were rarely observed (1,080 reads, 14 ASVs). All Dino-Group-V ASVs were of an undetermined family/group.
